# Expression and Polymorphism of Toll-Like Receptor 4 and Effect on NF-κB Mediated Inflammation in Colon Cancer Patients

**DOI:** 10.1371/journal.pone.0146333

**Published:** 2016-01-15

**Authors:** Abdelhabib Semlali, Narasimha Reddy Parine, Maha Arafah, Lamjed Mansour, Arezki Azzi, Omair Al Shahrani, Abdullah Al Amri, Jilani P. Shaik, Abdulrahman M. Aljebreen, Othman Alharbi, Majid A. Almadi, Nahla Ali Azzam, Muhammad Kohailan, Mahmoud Rouabhia, Mohammad Saud Alanazi

**Affiliations:** 1 Genome Research Chair, Department of Biochemistry, College of Science, King Saud University, Riyadh, Kingdom of Saudi Arabia; 2 College of Medicine, King Saud University, Riyadh, Kingdom of Saudi Arabia; 3 Division of Gastroenterology, King Khalid University Hospital, King Saud University, Riyadh, Kingdom of Saudi Arabia; 4 Division of Gastroenterology, The McGill University Health Center, Montreal General Hospital, McGill University, Montreal, Québec, Canada; 5 Groupe de Recherche en Écologie Buccale, Département de stomatologie, Faculté de Médecine Dentaire, Université Laval, Québec, Québec, Canada; 6 Department of Zoology, College of Science, King Saud University, Riyadh, Kingdom of Saudi Arabia; 7 College of Medicine, Al Imam Muhammad Ibn Saud Islamic University (IMSIU), Riyadh, Kingdom of Saudi Arabia; University of Kansas School of Medicine, UNITED STATES

## Abstract

Our aim was to evaluate the association between the expression and the polymorphism of TLR4/NF-κB pathways and colon cancer. TLR4 (*rs4986790*, *rs10759932*, *rs10759931* and *rs2770150*) were genotyped in blood samples from Colorectal patients and healthy controls. TLR4 and cytokines inflammatory expression were evaluated by real time PCR on 40 matching normal and colon tissues and the protein level by Immunohistochemistry. The high level of TLR4 expression in colon cancer tissues is mainly due to infections by bacteria in the human colon and leads to induction of an acute secretion of inflammatory cytokines mediated by NF-κB. Also, we report here a clear evidence for an association between TLR4 *rs10759931* polymorphism (OR = 0.086, CI: 0.04–0.18, P = <0.00001). This polymorphism affects the entire population without being specific to either gender or to any age group. In contrast, the *rs2770150* is associated with colon cancer in women aged over 50 years and is closely linked with the decreased levels of female sex hormones during the post-menopausal period (OR = 0.188, CI: 0.074–0.48, P = <0.00084). *rs10759932* and *rs4986790* appear to have any association with colon cancer. Our data suggest that TLR4 SNPs could possibly serve as biomarkers for decision making in colon cancer treatment.

## Introduction

Colorectal cancer (CRC) is a complex disease and one of the most common types of cancer. The risk factors for colon cancer appear to be determined by the interaction between genetics and a variety of environmental exposures. Several lines of evidence support the view that impairment of the immune system could contribute to the risk of developing many cancers, and the activation of the immune system is vigorously pursued for cancer therapy. In this sense, the association of innate immunity with colon cancer is emerging. Moreover, patients with immunodeficiency were shown to have an increased cancer risk [1], and recent studies have demonstrated that impaired signaling of innate immunity receptors may contribute to cancer pathogenesis [2–5]. Innate immunity is mainly activated and regulated by Toll-like receptors (TLRs) [6] found in the membranes of immune cells such as neutrophils, dendritic cells and macrophages as well as on cells of the skin surface and genitourinary and gastrointestinal tracts [7]. Toll-like receptor activation is responsible for the killing of microbial cells during infection while leaving the host cells intact [8–10]. Similar to other TLRs, TLR4 is composed of three domains: an extra-cellular domain comprising residues 24–631 and containing leucine-rich repeats (LRRs),a trans-membrane region with a single trans-membrane helix between residues 632–652, and a C-terminal cytoplasmic signaling domain [11, 12]. TLRs constitute a particularly important group of pattern recognition receptors (PRRs) [13] and are located on cell surfaces or within endosomes. These are predominantly expressed in tissues involved in immune function and have important roles in host defense against pathogenic organisms. To date, 10 functional TLRs have been described in humans; TLRs 11–13 are found in rodent species (rats and mice) [14].TLRs play a crucial role in the activation of the immune system by regulating the production of antiviral peptides and inflammatory cytokines [15–19]through highly conserved structural motifs known as pathogen-associated molecular patterns (PAMPs).TLRs have been implicated in innate immunity[5, 12, 20, 21], and alterations of TLR responses might result in severe viral and bacterial infections or a higher risk of cancer [4, 5, 20]; they have also been implicated in the inflammatory response of intestinal epithelial cells [22].

The signaling domains of TLRs are known as Toll IL-1 Receptor (TIR) domains because they share homology with the signaling domain of IL-1R family members [23], which induce NF-kB and IRF3 translocation and trans-activation [24] and the activation of different inflammatory cytokine genes [25, 26] by a MyD88-dependent pathway common to all TLRs, except TLR3 [27, 28]. Another TLR signaling mechanism known as the MyD88-independent pathway is associated with the stimulation of IFN-β and the maturation of dendritic cells through the adaptor TRIF/TICAM-1[29] or through TRAM/TICAM-2, which is restricted to the TLR4 pathway [30].

The TLR4 gene is located on chromosome9 and encodes a protein that functions in the immune response by activating nuclear factor kappa-light-chain-enhancer of activated B cells (NF-kB) [18]. The impairment of TLR signaling *in vivo* becomes evident through the presence of single-nucleotide polymorphisms(SNPs),which have been associated with receptor hypo-responsiveness and susceptibility to bacterial, fungal and viral infections[31]. A previous study highlighted the involvement of TLR activation and TLR polymorphisms in many diseases, such as prostate cancer [32]. Therefore, a decrease in the function of TLRs may be associated to an increase risk of cancer. It has been recently shown that a polymorphism in the promoter sequence of TLR2 and a TLR4 variant allele are involved in *Helicobacter pylori* (*H*. *pylori*)-associated gastric cancer [33, 34].In addition, a polymorphism in the TLR10-TLR1-TLR6 gene cluster is associated with an increased risk of prostate cancer [35], and functional TLR4 expression in vivo is known to be responsible for LPS-induced thrombocytopenia [36]. TLR-2 polymorphisms are also involved in areduced response after challenge with mycobacteria, whereasTLR4 polymorphisms are known to be associated with a reduced response to lipopolysaccharide and the development of gastric cancer [37, 38]. A TLR4 SNP (*rs11536898*) is also associated with colon cancer [39].Recently, a human TLR4 (Asp299Gly) SNP has been suggested to be more specifically associated with LPS hypo-responsiveness and an increased risk of developing prostate cancer among a North Indian population [40]. Additionally, TLRs have been demonstrated to play a role in the progression of polyps to tumors, and reduced TLR4 expression has been associated with the increased metastasis potential of colorectal cancer (CRC) [41, 42]. A synthetic meta-analysis based on data from 22 studies also confirmed the association of TLR4 SNP Asp299Gly with increased gastrointestinal cancer risk but with decreased prostate cancer risk [43]. However, a conflicting report of a multi-racial study conducted in Malaysia showed no correlation between a TLR4 polymorphism (Asp299Gly) and the risk of CRC[44]. Thus, it is clear that additional studies are necessary to validate these findings. The current study aims to evaluate the expression of TLR4 in CRC and to examine the potential implication of four SNPs of human TLR4 (*rs32770150*, *rs310759931*, *rs10759932and rs4986790*) with the risk of colon cancer in the Saudi Arabia population.

## Results

### Analysis of clinical data parameters

A total of 115 colon cancer cases and 102 healthy controls were included in the study. The clinical characteristics of the patients, including age, nationality, family history, smoking habits, and stage of colon cancer, were collected and compared with those of the control patients (Table 1). The study population age ranged between 45 and 88years, with a mean age of 56.04 ± 14.37 for the colon cancer cases and 52.84 ± 15.88 for the controls. There was no significant difference in the mean age between the two groups. The male to female ratio was not significantly different between the cases and controls (66/49 for patients and 60/42 for controls).

**Table 1:** Clinical characteristics of the subjects for genotyping.

Characteristics	Cancer (115)	Control (102)	p value
**Gender**			
Male	66	60	0.59
Female	49	42	0.46
**Age**	56.04 ± 14.37	52.84 ± 15.88	0.77
**Tumor Location**			
Colon	76	-	
Rectum	39	-	
**Smoking Status**			
Smoker	7	5	0.56
Nonsmoker	108	97	0.44
**Alcohol habit**			
Alcoholic	2	-	
Nonalcoholic	113	-	
**Treatment**			
Under Chemotherapy	3	-	
No Chemotherapy	112	-	
Under Radiotherapy	5	-	
No Radiotherapy	110	-	

### Increased TLR4 gene expression in colon cancer tissues compared to matching normal tissues

**Fig 1:**
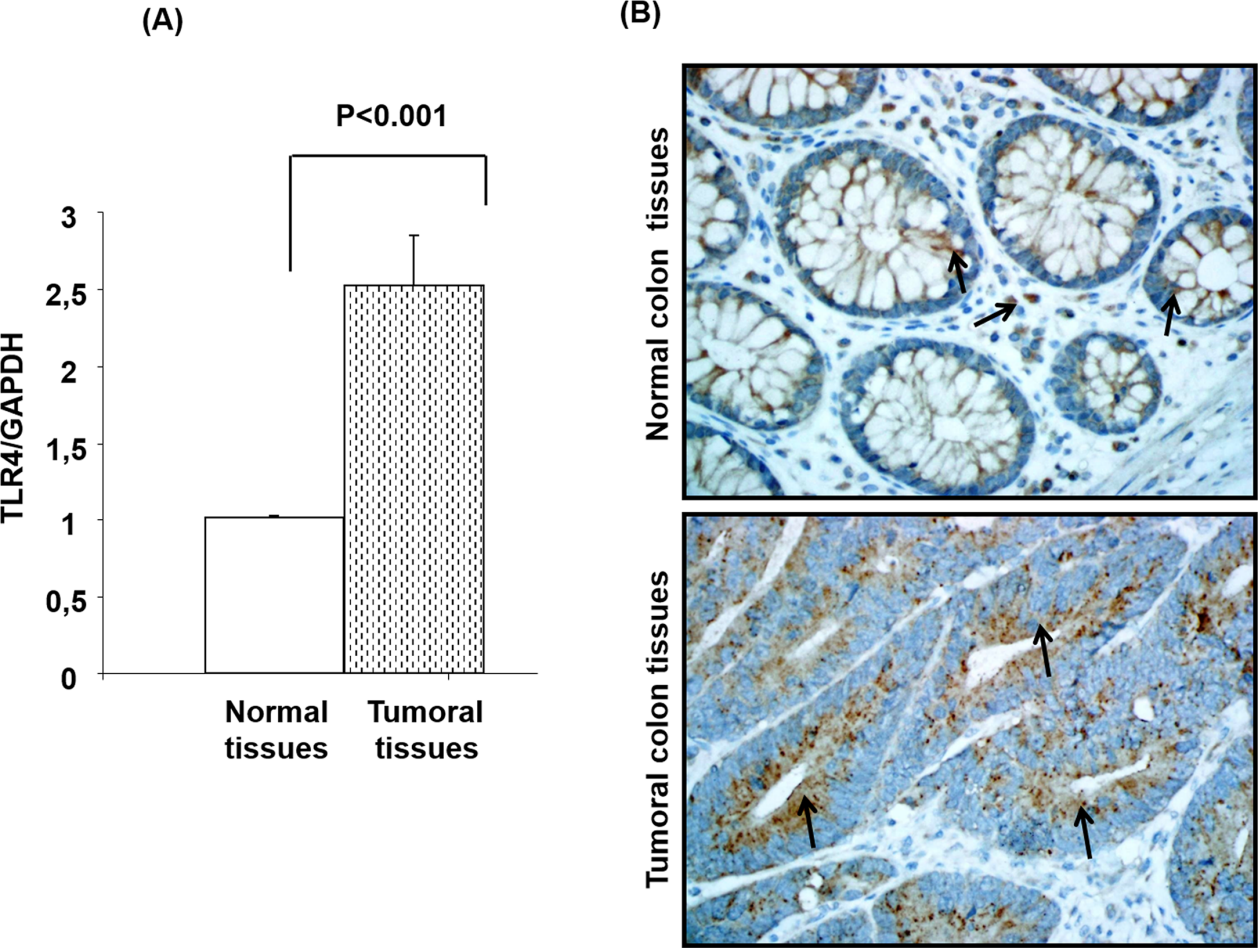
Toll-Like-Receptor 4 (TLR4) mRNA and protein expression in colon cancer tissues. Total cellular RNA freshly extracted from matching normal and colon cancer tissues was reverse transcribed into cDNA and then used to measure the TLR4mRNA expression (Panel A). Tissues were immunostained using specific TLR4 antibody (Panel B).

The tissue samples used for RNA analysis (n = 40 matching tissues) were immediately stored in RNA-later solution. After RNA extraction, a quantitative real-time reverse transcription PCR was performed to compare mRNA differential expression of TLR4. As shown in Fig 1A, TLR4 was highly expressed (p <0.001) in colon cancer tissues compared to normal colon tissues (2.53 ± 0.32) and (1.01 ± 0.01) respectively (**Fig 1A)**.

To confirm the mRNA expression data, we determined the TLR4 protein expression by immunohistochemistry. As shown in **(Fig 1B),** positive immuno-staining for the TLR4 was generally observed in colon epithelial cells and also in some stromal compartments cells. However, the intensity of staining was higher in the adenocarcinoma colon tumor tissues.

### Correlation between TLR4 polymorphisms and susceptibility to colon cancer development in Saudi Arabian patients

The distribution of alleles and genotypes and the association analysis are described in Table 2. All SNPs genotyped were tested for the Hardy–Weinberg equilibrium (HWE). DNA samples from the blood of the 115 patients and 102 healthy controls were genotyped for the presence of 4 TLR4 SNPs (*rs2770150* T/C, *rs10759931*A/G, *rs10759932*T/C and *rs4986790*A/G). The phenotypic and genotypic characteristics of the patient and control groups are summarized in Table 3.

**Table 2:** Frequencies of TLR 4 gene polymorphism in colorectal cancer and controls.

Polymorphism	Patients%	Control%	OR	CI	X^2^	P value	cP value*
**rs2770150**							
T	163 (0.7)	128 (0.63)	Ref				
C	74 (0.3)	76 (0.37)	0.692	0.463–1.035	3.23	0.07230	0.2892
T/T	60(0.52)	42 (0.41)	Ref				
T/C	43 (0.38)	44 (0.43)	0.684	0.384–1.217	1.67	0.19594	0.78376
C/C	12 (0.1)	16(0.16)	0.525	0.225–1.223	2.27	0.13220	0.5288
T/C + CC	55 (0.48)	60 (0.59)	0.642	0.375–1.099	2.62	0.10523	0.42092
**rs10759931**							
G	190(0.87)	59(0.3)	Ref				
A	26(0.13)	139(0.70)	0.058	0.035–0.097	145.80	**<0.00001**	**<0.00001**
GG	88 (0.81)	10 (0.10)	Ref				
GA	14 (0.13)	39 (0.4)	0.041	0.017–0.100	63.04	**<0.00001**	**<0.00001**
AA	6 (0.56)	50 (0.50)	0.014	0.005–0.040	93.71	**<0.00001**	**<0.00001**
GA + AA	20 (0.69)	89 (0.9)	0.026	0.011–0.058	105.57	**<0.00001**	**<0.00001**
**rs10759932**							
T	36 (0.16)	28 (0.14)	Ref				
C	192(0.84)	176 (0.86)	0.848	0.497–1.448	0.36	0.54660	1
TT	3(0.03)	1 (0.01)	Ref				
TC	30 (0.27)	26 (0.25)	0.385	0.038–3.927	0.69	0.40527	1
CC	81 (0.70)	75 (0.74)	0.360	0.037–3.537	0.83	0.36145	1
TC + CC	111 (0.97)	101 (0.99)	0.366	0.038–3.579	0.81	0.36885	1
**rs4986790**							
A	219(0.96)	191(0.95)	Ref				
G	9 (0.04)	9(0.45)	1.090	0.424–2.799	0.03	0.85856	1
GG	1(0.008)	1(0.01)	Ref				
AG	7 (0.062)	7(0.07)	1.000	0.052–19.36	0.005	0.98	1
AA	106 (0.93)	92(0.92)	1.093	0.067–17.71	0.004	0.95020	1
AG+GG	8 (0.07)	8 (0.08)	1.087	0.067–17.59	0.004	0.95340	1

*cP value: Bonferroni corrected P value.

**Table 3:** Distribution of TLR-4 SNPs genotype and allele frequencies in colorectal cancer cases and control population based on gender (Female).

Polymorphism	Patients %	Control %	OR	CI	X^2^	P value	cP value*
**rs2770150**	(n = 35)	(n = 31)					
T	78 (0.78)	49 (0.6)	Ref				
C	22 (0.22)	33 (0.4)	0.188	0.074–0.480	13.75	**0.00021**	0.00084
T/T	31 (0.89)	16 (0.51)	Ref				
T/C	1 (0.024)	7 (0.23)	0.074	0.008–0.653	8.03	**0.00460**	0.0184
C/C	3 (0.086)	8 (0.26)	0.194	0.045–0.831	5.50	**0.01902**	0.07608
T/C + CC	4 (0.11)	15 (0.48)	0.138	0.039–0.484	10.95	**0.00093**	0.00372
**rs10759931**	(n = 46)	(n = 38)					
G	81 (0.88)	22 (0.29)	Ref				
A	11 (0.12)	54 (0.71)	0.055	0.025–0.123	61.27	**<0.00001**	**<0.00001**
GG	38 (0.82)	4 (0.1)	Ref				
GA	5 (0.11)	14 (0.38)	0.038	0.009–0.160	25.89	**<0.00001**	**<0.00001**
AA	3 (0.07)	20 (0.52)	0.016	0.003–0.078	38.26	**<0.00001**	**<0.00001**
GA + AA	8 (0.17)	34 (0.98)	0.025	0.007–0.090	43.25	**<0.00001**	**<0.00001**
**rs10759932**	(n = 48)	(n = 40)					
T	16 (0.16)	8 (0.1)	Ref				
C	80 (0.84)	72 (0.9)	0.556	0.224–1.375	1.65	0.19940	0.7976
TT	2 (0.04)	0 ()	Ref				
TC	12 (0.25)	8 (0.2)	0.294	0.012–6.925	1.26	0.26219	1
CC	34 (0.71)	32 (0.8)	0.212	0.010–4.592	1.83	0.17593	0.70372
TC + CC	46 (0.96)	40 (1)	0.230	0.011–4.924	1.71	0.19158	0.76632
**rs4986790**	(n = 54)	(n = 44)					
A	96 (0.96)	80 (0.98)	Ref				
G	4 (0.04)	2 (0.02)	1.667	0.298–9.337	0.34	0.69256	1
AA	46 (0.92)	39 (0.95)	Ref				
AG	4 (0.08)	2 (0.05)	1.696	0.295–9.759	0.36	0.55045	1
GG	0 (0)	0 (0)	0.849	0.016–43.8	0	1	1
AG+GG	4 (0.08)	2 (0.05)	1.696	0.295–9.759	0.36	0.55045	1

*cP value: Bonferroni corrected P value.

The data for three TLR4 SNPs, *rs2770150*, *rs10759932* and *rs4986790*, which are located in the promoter, did not show any association with colon cancer in the Saudi population. However, for SNP *rs10759931*, which results in Asp299Gly, the distribution of alleles and genotypes were significantly different between the patients and controls. The G allele was found to be significantly more highly frequent among the patients (0.87) compared to the control group (0.3). A homozygous GG of the same SNP was also highly frequent in the patient group compared to the control group (0.81 *vs*. 0.1) and was highly associated with the development of colon cancer among the Saudi population. However, the presence of the allele A in the case of heterozygotes and homozygous (AA) appears to confer protection against colorectal cancer (OR 0.026; 95% CI 0.011–0.058; p ≤0.00001).

### Association between the genotype frequencies of TLR4 gene polymorphisms and gender

To evaluate the association between colon cancer risk and individual SNPs based on a patient’s gender, the genotype distributions according to gender in the colon cancer group were compared with those of the control subjects (Tables 3 and 4). Interestingly, in the female group (35 patients and 31 controls), the T allele of SNP rs2770150 presented a frequency that was significantly higher in the patients than in the controls (0.78 versus 0.6) (OR 2.38; 95%CI 1.2–4.8 and p = 0.012). Conversely, genotypes TC and CC were four times less frequent in the cases compared to the controls (OR 0.14; 95%CI 0.04–0.48 and p = 0.0009). Allele (C) was significantly highly frequent among the controls (0.40) compared to the patient group (0.22) (p = 0.0002). However, the distribution of the CC genotype between affected and non-affected females was different (p = 0.019) (Table 3). SNP *rs2770150* CC genotype didn’t showed significant risk in female patients after applying bonferroni’s correction. In contrast, SNP *rs2770150* was not associated with colorectal cancer in males (40 patients and 34 controls (Table 4).

**Table 4:** Distribution of TLR-4 SNPs genotype and allele frequencies in colorectal cancer cases and control population based on gender (Male).

Polymorphism	Patients %	Control %	OR	CI	X^2^	P value	cP value*
**rs2770150**	(n = 40)	(n = 34)					
T	85 (0.65)	71 (0.65)	Ref				
C	45 (0.35)	39 (0.35)	0.926	0.442–1.939	0.04	0.83828	1
T/T	29 (0.72)	24 (0.7)	Ref				
T/C	2 (0.06)	2 (0.06)	0.828	0.108–6.322	0.03	0.85508	1
C/C	9 (0.22)	8 (0.24)	0.931	0.311–2.784	0.02	0.89824	1
T/C + CC	11 (0.37)	10 (0.3)	0.910	0.331–2.507	0.03	0.85575	1
**rs10759931**	(n = 62)	(n = 48)					
G	109 (0.88)	32 (0.3)	Ref				
A	15 (0.12)	76 (0.7)	0.051	0.025–0.103	84.42	**<0.00001**	**<0.00001**
GG	50 (0.8)	6 (0.13)	Ref				
GA	9 (0.15)	14 (0.29)	0.077	0.023–0.254	21.69	**<0.00001**	**<0.00001**
AA	3 (0.05)	28 (0.58)	0.013	0.003–0.055	53.12	**<0.00001**	**<0.00001**
GA + AA	12 (0.2)	42 (0.87)	0.034	0.012–0.099	50.27	**<0.00001**	**<0.00001**
**rs10759932**	(n = 64)	(n = 55)					
T	18 (0.14)	17 (0.15)	Ref				
C	110 (0.86)	93 (0.85)	1.117	0.545–2.291	0.09	0.76241	1
TT	1 (0.02)	1 (0.02)	Ref				
TC	16 (0.25)	15 (0.27)	1.067	0.061–18.624	0.003	0.96472	1
CC	47 (0.73)	39 (0.71)	1.205	0.073–19.9	0.02	0.89610	1
TC + CC	63 (0.98)	54 (0.98)	1.167	0.071–19.1	0.01	0.91385	1
**rs4986790**	(n = 64)	(n = 54)					
A	123 (0.96)	103 (0.95)	Ref				
G	5 (0.04)	5 (0.05)	0.837	0.236–2.973	0.08	1.00002	1
AA	60 (0.93)	50 (0.92)	Ref				
AG	3 (0.05)	3 (0.06)	0.833	0.161–4.312	0.05	0.82770	1
GG	1 (0.02)	1 (0.02)	0.833	0.051–13.66	0.02	0.89821	1
AG+GG	4 (0.06)	4 (0.07)	0.833	0.198–3.503	0.06	0.80324	1

*cP value: Bonferroni corrected P value.

SNP *rs10759931*, which showed a high association with colon cancer risk in the overall population, also presented a significant association in the female (46 patients and 38 controls) and male (62 patients and 48 controls) sub groups. However, genotypes GA and AA in the female group were frequent in the control samples compared to the colon cancer patients (OR = 0.025 95% CI 0.007–0.090 and p < 0.00001; Table 3). The same observation was made in the male patients compared to the healthy males: GA+AA were 0.2 and 0.87, respectively (p< 0.00001; Table 4). For SNPs*rs10759932* and r*s4986790*, no significant difference in the distribution of genotypes between affected and non- affected females and males was observed (p > 0.05) (Tables 3 and 4).

### Association between genotype frequencies of TLR4 gene polymorphisms and age

Further analysis of the TLR4 genotype distribution after correlation with age revealed that the median age of onset of present colon cancer samples is 56 years and in control groups is 52 years. To evaluate the association between TLR4 SNPs with a younger age at diagnosis of colon cancer, we stratified the patients as ≤50 or >50 years of age. The genotype distribution for the individual SNPs along with the statistical analysis is shown in Tables 5 and 6.The T allele of SNP rs2770150 presented a frequency that was significantly higher in the patients over 50 years of age than in the controls. Conversely, the C allele was significantly less frequent in the cases compared to the controls (0.24 versus 0.36) (OR 0.57; 95%CI 0.3–0.9 and p = 0.038) (Table 6), whereas no difference was observed for this SNP for the subpopulation of patients aged less than 50 (Table 5). But after applying bonferroni’s correction SNP rs2770150 CC genotype didn’t showed significant risk in >50 year old patients.rs10759931, which showed a significant association in the overall study, also indicated a significant risk in both subpopulations of patients (p< 0.00001) (Tables 5 and 6). No significant difference in the distribution of genotypes between affected and non-affected individuals for both subpopulations was observed (p > 0.05) for the other two SNPs (*rs10759932* and *rs4986790*; Tables 5 and 6).

**Table 5:** Distribution of TLR-4 SNPs genotype and allele frequencies in colorectal cancer cases and control population based on age (≤ 50 years).

Polymorphism	Patients %	Control %	OR	CI	X^2^	P value	cP value*
**rs2770150**	(n = 38)	(n = 40)					
T	46 (0.6)	49 (0.61)	Ref				
C	30 (0.4)	31 (0.39)	1.031	0.542–1.962	0.01	0.92623	1
T/T	15 (0.4)	16 (0.4)	Ref				
T/C	16 (0.42)	17 (0.42)	1.004	0.376–2.677	0.00	0.99376	1
C/C	7 (0.18)	7 (0.18)	1.067	0.302–3.770	0.01	0.92018	1
T/C + CC	23 (0.55)	24 (0.6)	1.022	0.413–2.533	0.00	0.96213	1
**rs10759931**	(n = 37)	(n = 40)					
G	61 (0.82)	23 (0.29)	Ref				
A	13 (0.18)	57 (0.71)	0.086	0.040–0.186	44.68	**<0.00001**	**<0.00001**
GG	27 (0.73)	5 (0.12)	Ref				
GA	7 (0.19)	13 (0.33)	0.100	0.027–0.375	13.26	**0.00027**	0.00108
AA	3 (0.08)	22 (0.55)	0.025	0.005–0.118	29.49	**<0.00001**	**<0.00001**
GA + AA	10 (0.27)	35 (0.87)	0.053	0.016–0.173	28.94	**<0.00001**	**<0.00001**
**rs10759932**	(n = 37)	(n = 40)					
T	9 (0.12)	11 (0.14)	Ref				
C	65 (0.88)	69 (0.86)	1.151	0.448–2.959	0.09	0.76963	1
TT	0 (0)	1 (0.03)	Ref				
TC	9 (0.24)	9 (0.22)	3.000	0.108–83.36	0.95	0.32972	1
CC	28 (0.76)	30 (0.75)	2.803	0.11–71.656	0.92	0.33779	1
TC + CC	37 (1)	39 (0.97)	2.848	0.11–72.118	0.94	0.33301	1
**rs4986790**	(n = 38)	(n = 38)					
A	74 (0.97)	72 (0.95)	Ref				
G	2 (0.03)	4 (0.05)	0.486	0.086–2.739	0.69	0.68322	1
AA	37 (0.97)	35 (0.92)	Ref				
AG	0 (0)	2 (0.05)	0.189	0.009–4.082	2.06	0.15165	1
GG	1 (0.03)	1 (0.03)	0.946	0.057–15.71	0.00	0.96908	1
AG+GG	1 (0.03)	3 (0.08)	0.315	0.031–3.176	1.06	0.30423	1

*cP value: Bonferroni corrected P value.

**Table 6:** Distribution of TLR-4 SNPs genotype and allele frequencies in colorectal cancer cases and control population based on age (> 50 years).

Polymorphism	Patients %	Control %	OR	CI	X^2^	P value	cP value*
**rs2770150**	(n = 77)	(n = 53)					
T	117 (0.76)	68 (0.64)	Ref				
C	37 (0.24)	38 (0.36)	0.566	0.329–0.973	4.28	**0.03866**	0.15464
T/T	45 (0.58)	23 (0.43)	Ref				
T/C	27 (0.35)	22 (0.42)	0.627	0.295–1.334	1.48	0.22445	0.8978
C/C	5 (0.07)	8 (0.15)	0.319	0.094–1.088	3.55	0.05960	0.2384
T/C + CC	32 (0.42)	30 (0.56)	0.545	0.269–1.106	2.85	0.09146	0.36584
**rs10759931**	(n = 37)	(n = 49)					
G	129 (0.91)	28 (0.29)	Ref				
A	13 (0.09)	70 (0.71)	0.085	0.041–0.179	48.99	**<0.00001**	**<0.00001**
GG	27 (0.73)	4 (0.08)	Ref				
GA	7 (0.19)	20 (0.4)	0.052	0.013–0.202	22.26	**<0.00001**	**<0.00001**
AA	3 (0.08)	25 (0.52)	0.018	0.004–0.087	34.34	**<0.00001**	**<0.00001**
GA + AA	10 (0.27)	45 (0.92)	0.033	0.009–0.115	38.41	**<0.00001**	**<0.00001**
**rs10759932**	(n = 77)	(n = 47)					
T	27 (0.18)	14 (0.13)	Ref				
C	127 (0.82)	90 (0.87)	0.498	0.223–1.112	2.98	0.08435	0.3374
TT	3 (0.04)	0 (0)	Ref				
TC	21 (0.27)	9 (0.19)	0.323	0.015–6.895	1.24	0.26595	1
CC	53 (0.69)	38 (0.81)	0.199	0.010–3.956	2.10	0.14703	0.58812
TC + CC	74 (0.96)	47 (1)	0.224	0.011–4.435	1.88	0.17072	0.68288
TLR4 rs4986790	(n = 76)	(n = 52)					
A	145 (0.95)	101 (97)	Ref				
G	7 (0.05)	3 (0.03)	1.625	0.410–6.436	0.49	0.74439	1
AA	69 (0.91)	49 (0.94)	Ref				
AG	7 (0.09)	3 (0.06)	1.657	0.408–6.727	0.51	0.47615	1
GG	0 (0)	0 (0)	0.712	0.014–36.506	0	1	1
AG+GG	7 (0.09)	3 (0.06)	1.657	0.408–6.727	0.51	0.47615	1

*cP value: Bonferroni corrected P value.

### Inflammatory Innate Molecules Response in relation to colon cancer development

**Fig 2:**
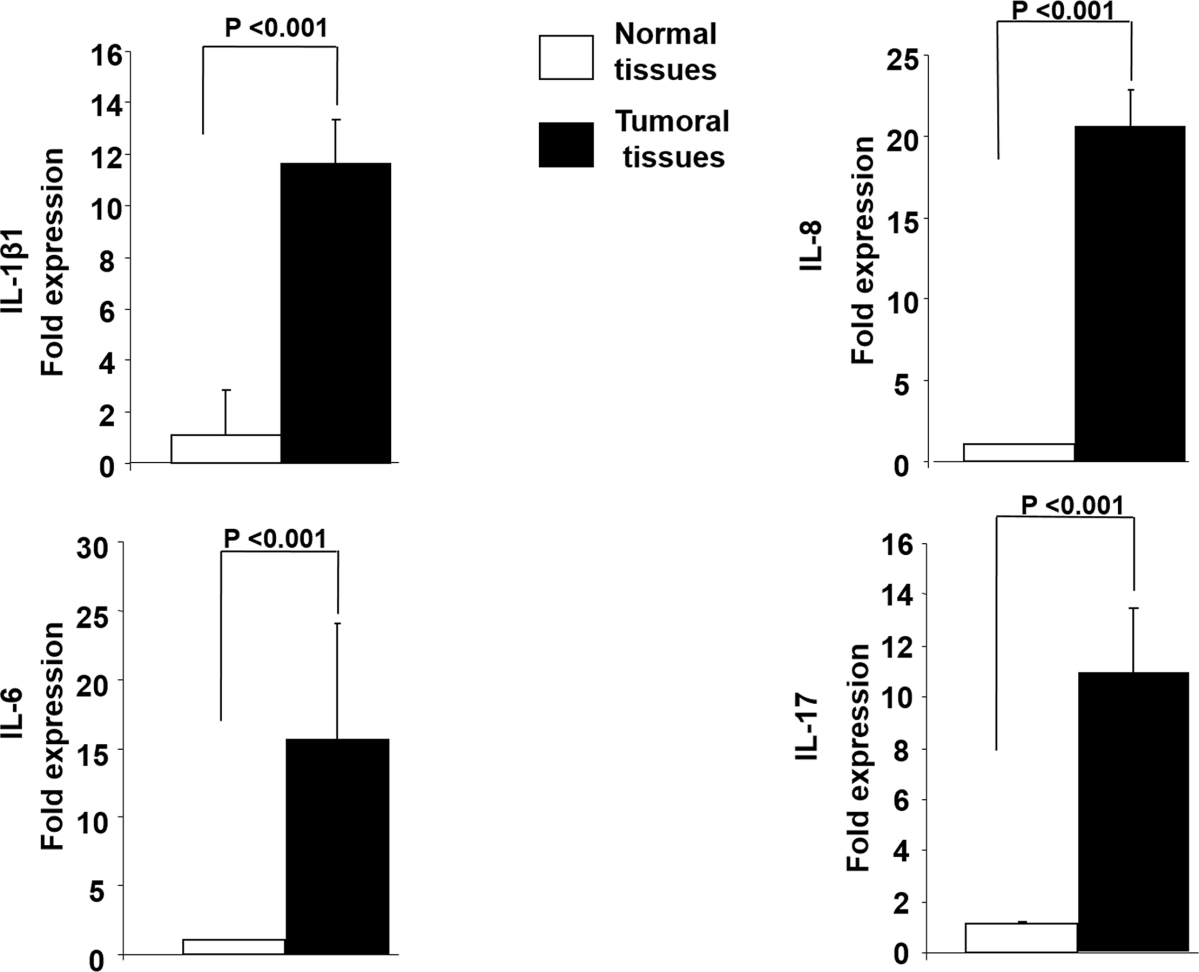
Inflammatory cytokines expression in colon cancer tissues compared to normal matching tissues: Cytokine production was assessed by qRT-PCR on colonic cancer tissues and matching normal tissues (mean ± SD, n = 40 patients, data normalized to *GAPDH*, control (open bars), cancer (solid bars), *P<0.00 t-test)

The aim of the present study was to evaluate the differences and relationships between the expression of TLR4 and cytokines, specifically variations in expression of inflammatory cytokines interleukin (IL1β1, IL6, IL-8 and IL-17. The presence of inflammatory cells and expression levels of key cytokines involved in the colon cancer inflammation process were quantified by using real-time reverse transcriptase (RT)-PCR from 40 colon cancer tissues and 40 normal matching tissues. The mRNA of cytokines was substantially higher levels (10–22-fold differences) in colon cancer tissues compared to normal colon samples, as shown in **Fig 2**, indicating higher pro-inflammatory responses in CRC samples. This is due to overexpression of TLR4 activity in colon cancer patients.

### Increased NF-κB p65 expression in colon cancer tissues

**Fig 3:**
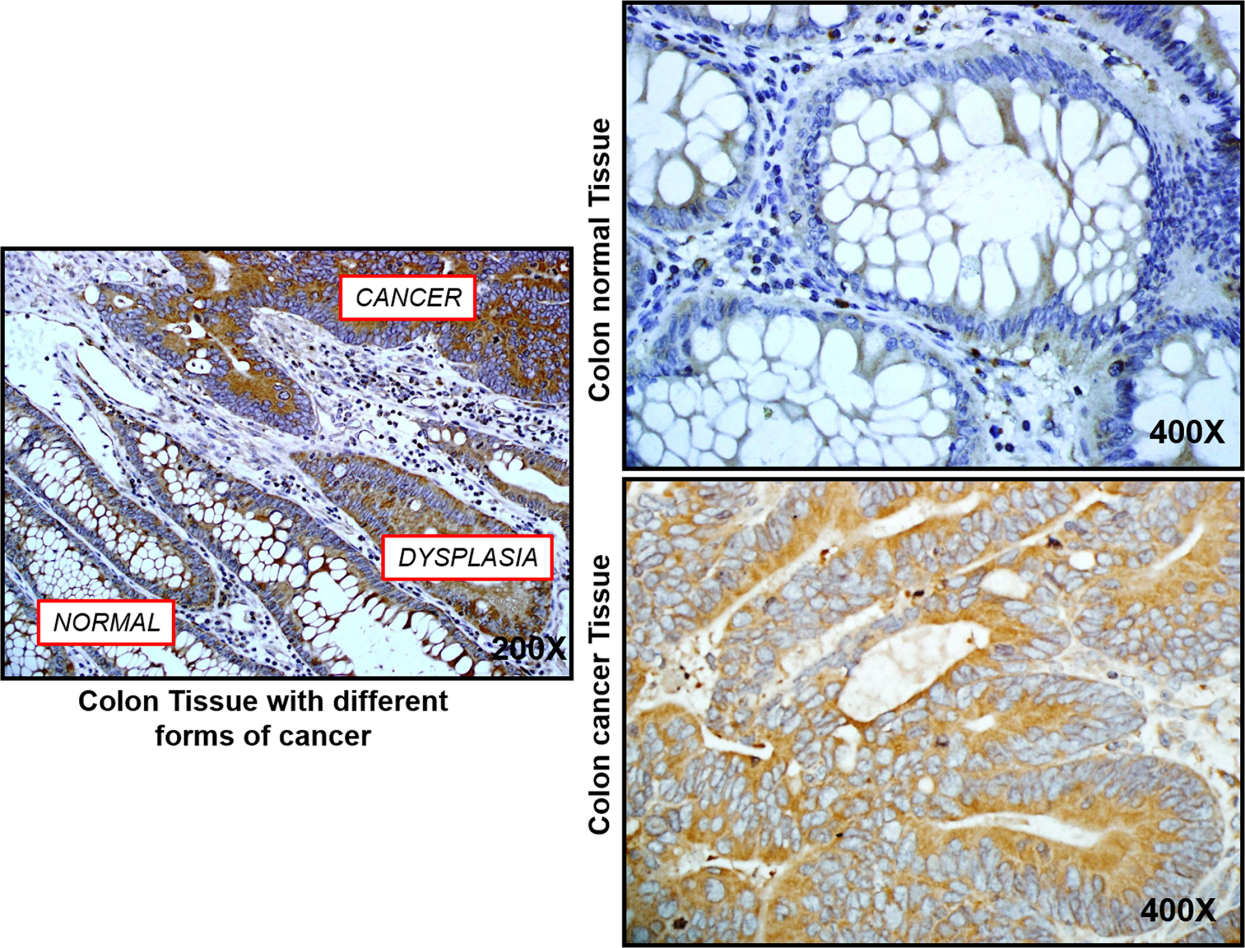
NF-κB expression in colon cancer tissues. Tissues were immunostained using specific NF-kB antibody (p65) at 1/100 (n = 10).

To confirm our hypothesis that, the highly level of TLR4 in colon cancer patients leads to induction of an inflammatory response mediated by multiple pathways specifically NF-κB and induce an acute secretion of inflammatory cytokines as such as IL-6, a comparative study of NF-κB expression was performed by immunohistochemistry on cancer colon tissues and normal matching tissues. Immunohistochemistry analysis revealed that NF-κB p65 expression in colon cancer tissue increased significantly compared to normal colon tissue (**Fig 3**).Our results suggest a critical role of NF-κB as a signaling molecule in inflammatory process, which facilitates the expression and secretion of pro-inflammatory cytokines, leading to a series of inflammatory responses by TLR4 activation.

## Discussion

The association between inflammation and carcinogenesis was first described 10 years ago[45–47].Since then, inflammation has been reported to increase proliferation and migration/invasion in various types of tumor cells [47], and the link between commensal microorganism-induced inflammation of the mucosa has recently been suspected as being linked to CRC. Conversely, our hypothesis is that TLR4 plays an important role in the innate immune system and that alteration of the structural function of this protein, which ultimately hampers immune surveillance in colon mucosa, may lead to tumor development [48]. It is documented that TLR4 activation promotes carcinogenesis and resistance to chemical treatments in breast cancer [49, 50], whereas blocking TLR4 activation can slow breast cancer growth and prolong survival [2]. Therefore, we first examined association between the susceptibility to develop CRC in the Saudi population and TLR4 expression and TLR4 polymorphism. Our results show that TLR4 is over-expressed in colon cancer tumor epithelial cells, which constitute the first line of defense against foreign agents. Moreover, our analysis also demonstrated that TLR4 expression is significantly increased in colon tumor tissues compared with matching normal colon tissues. These results are in agreement with previous work reporting the over-expression of TLR4in various cancers, such as gastric cancer progression [51], breast cancer[49, 50, 52], colon cancer [52], and ovarian cancer[53]. The higher level in TLR4 expression in colon cancer tissues compared to normal tissues is mainly due to infections by bacteria in the human colon, explaining a potential origin of colon cancer. These findings suggest that the activation of TLR4 expression in colon cancerous tissues may promote tumor growth and resistance to apoptosis, a conclusion that is supported by the previous study that showed that the blockage of TLR4 signaling delays tumor growth and prolongs the survival of animals [41, 54]. TLR4 signaling in cancer is considered a double-edged sword: if TLR4 is activated on immune cells, it can enhance anti-tumor immunity, and thus TLR4 could be used as a marker for the detection of a predisposition to a cancer; however, chronic inflammation induced by TLR4 activation is a major risk factor for cancer development [55].TLR4 could be a major player in colon cancer development and impairment inTLR4 signaling in vivo becomes evident through the presence of single-nucleotide polymorphisms(SNPs).

This is the first report examining a possible association of several commonTLR4 polymorphisms with colon cancer in Saudi Arabians. Given the higher frequency of the *TLR4* G allele in CRC patients compared to healthy controls, clear evidence is presented for a novel association between TLR4*rs10759931*polymorphism and susceptibility to colon cancer development in the Saudi Arabian population. The *TLR4*GG genotype is the most widespread in the Middle East, particularly in the Saudi Arabian population. This polymorphism affects the entire population without being specific to either gender or any age group. The AA genotype is generally represented in other populations and is protective against colon cancer thus the GG genotype might serve as a genetic marker for the diagnosis of colon cancer in this ethnic population. In contrast, the*TLR4*polymorphism *rs2770150*is associated with colon cancer in women aged over 50 years and is closely linked with the decreased levels of female sex hormones during the post-menopausal period. Previous studies have already described the role of the estrogen and progesterone in protecting against colon cancer in women [56–59]. In this sense, we investigated this polymorphism in pre- and post-menopausal women to help clarify this phenomenon.

The most common missense polymorphism, D299G, which occurs in exon 4 of the human TLR4 gene (A896G), is located within the extracellular domain of the receptor. The X-ray structures of both wild-type and mutated (D299G) receptors with LPS and the MD-2 domains have been determined [60], clearly showing that this polymorphism does not affect dimerization but does result in a significant local conformational change at the D299G site [61]. This conformational change may impair ligand binding and lower the receptor’s signaling response to LPS, and impaired TLR4 signaling has been shown to interfere with the recruitment of MyD88 and TRIF[62], which would lead to a depressed immune response to any commensal bacterial aggression in mucosa cells. This polymorphism together with TLR4rs2770150 showed no association with colon cancer in this ethnic group, even though these two SNPs showed a strong link with prostate cancer [32] and gastric cancer[63]in other studies and in other populations.

TLR4 Activation by ligands leads to cytokine production that promotes a major role in the pathogenesis of same cancer, especially in initiation and maintenance of inflammation. The role of cytokines such as IL-6 plays important roles in with a wide range of biological activities in immune hematopoies is regulation and oncogenesis. The majority of these cytokines has also been detected in multiple epithelial tumors [64],is involved in the proliferation and differentiation of various malignant tumor cells[65]. Overexpression of IL-6 has been found in prostate cancer [66, 67], breast carcinoma[68]and oral squamous cell carcinoma[69].

Here we show enhanced expression of TLR-4 on colon cancer tissues and its role in the inflammatory pathways particularly via NF-κB pathway, a fundamental molecular hub linking inflammation and cancer. Inhibiting the activation of NF-κB by blocking the MyD88 signal will reduce the release of proinflammatory cytokines, alleviate the inflammatory response and achieve a therapeutic effect.

**In summary**, we provide links between TLR4 gene expression and TLR4 polymorphisms, which have been hypothesized as important to the carcinogenic process of colon cancer in the Saudi Arabia population. Our data suggest that genetic variation in TLR4 may influence the development of colon cancer. Replication of these findings is warranted given the biological plausibility of the association and the interaction with diet and lifestyle factors that can modify these genetic effects. Furthermore, we believe that our study provides an important mechanistic link that could be the starting point for further studies, and important SNPs in this genes could possibly serve as biomarkers for decision making in colon cancer treatment.

## Materials and Methods

### Study populations and sample collection

A total of 115 colon cancer cases and 102 healthy controls were included in this study, which was approved by the local institutional review board. The samples were recruited from King Khalid University Hospital in Riyadh, Saudi Arabia. All questionnaire data and (tissue and blood) samples were collected at the initial recruitment of both the cases and controls. The participants were asked to complete a self-administered questionnaire regarding their socio-demographic characteristics (e.g., age, family history of cancer), lifestyle (e.g., smoking habits and alcohol intake), and personal medical history. The cases and controls were frequency-matched by age and sex. The clinical characteristics of the patients, including age for the selection of the Saudi population(non-Saudi citizen individuals were excluded), family history, smoking habits, stage of colon cancer, medications and presence of other diseases were collected and compared. The study population age ranged between 45 and 88years, with a mean age of 57.04 ± 14.37 for the colon cancer cases and 56.51± 15.70 for the controls (Table 1). The tissue samples were used for RNA extraction. The blood samples were stored in EDTA tubes at -80°C and used for genomic DNA extraction.

### General reagents

DNA/primers and SNPs were obtained from Life Technologies/ Invitrogen (Burlington, ON, Canada). Anti-TLR4 antibodies were purchased from Santa Cruz Biotechnology, Inc (Santa Cruz, CA,USA).RNA later for RNA stabilization was obtained from Ambion / Life Technologies (Burlington, ON, Canada). RNA kits were from Qiagen (Hilden, Germany). The high-capacity cDNA reverse transcription kit was from Applied Biosystems (Warrington, USA). Sybr Green was obtained from Bio-Rad (Mississauga, ON, Canada).

### DNA extraction

Genomic DNA was extracted from whole blood using the QIAmp kit (QIAmp DNA Blood Mini Kit, Qiagen, Valencia, CA, USA) according to the manufacturer’s instructions. Briefly, 200 to 300 μl of blood stored in EDTA tubes at -80°C was equilibrated at room temperature, mixed with protease and lysis buffer, and incubated at 56°C for 10 minutes. Then, 100%ethanol was added, and the mixture was passed through the column using centrifugation. The column membrane was washed, and the DNA was eluted with100 μl elution buffer (AE). The concentration of extracted DNA was determined using a Nano-Drop8000spectrophotometer(Thermo Scientific).The purity of DNA was evaluated by standard A260/A280 and A260/A230 ratios.

### Total RNA isolation

Total tissue RNA was isolated using the DNA/RNA Mini kit (Qiagen, Hilden, Germany), as described by Alanazi et al [70]. Briefly, tissues were lysed in 350 μllysis buffer with 3.5 μl β-mercaptoethanol. The lysate was subsequently filtered and homogenized with 70% ethanol, and the DNA was loaded onto the column and digested with DNase at room temperature for 30 minutes. Afterwards, the column membrane was washed, and the RNA was eluted with 40μl RNase-free H_2_O and stored in nuclease-free collection tubes at -80°C. The concentration, purity, and quality of the isolated RNA were all determined using the Agilent 2100 Bio analyzer system and Agilent Small RNA analysis kit according to the instructions provided by the manufacturer (Agilent Technologies, Waldbronn, Germany).

### cDNA synthesis

As described by Semlali et al [71, 72], 1μg of RNA each sample was reverse transcribed into cDNA using a high-capacity cDNA reverse transcription kit (Applied Biosystems, Warrington, USA).The conditions for the preparation of the cDNA templates for PCR analysis were 10 min at 25°C, 2 h at 37°C, and 5 min at 85°C. The synthesized cDNA was stored at -20°C or 4°C for subsequent PCR reactions.

### Quantitative real-time PCR

TLR4 mRNA expression was detected by RT-qPCR as previously described [71, 72]. The expression of the house-keeping gene GAPDH was used as an endogenous control for normalization of the amount of sample RNA. The reactions were performed using a PCR SYBR Green Supermix from Applied Biosystems. The sequences of TLR4, cytokines and GAPDH primers are shown in S1 Table.

Real-time PCR was performed using a7500 Real-Time PCR System (Applied Biosystems). The primers were added to the reaction mix at a final concentration of 250 nM. As previously described[72], 5μlof each cDNA sample was added to a 20-μl PCR mixture containing 12.5 μl of SYBR Green Supermix,0.5 μl of specific primers and 7 μl of RNase-/DNase-free water. The PCR reaction consisted of with 50°C for 2 minutes and 95°C for 5 minutes, followed by 40 cycles of 95°C for 15 seconds,62°Cfor30 minutes and 30 seconds at 72°C. All real-time PCR assays were performed in triplicate, and the specificity of each primer pair was verified by the presence of a single melting temperature peak. GAPDH produced uniform expression levels varying by less than 0.5 CTs between sample conditions and was therefore used as a reference gene for this study. The amplified products were separated on an agarose gel to confirm that there were no spurious products amplified. The results were analyzed using the 2^-ΔΔCt^ (Livak) relative expression method.

### Immunohistochemistry (IHC) analysis

Paraffin-embedded blocks of colon cancer and normal colon tissue specimens were cut into 3-μm-thick sections. These were mounted on saline-coated slides and incubated for 15 to 20 minutes in a hot air oven at 60°C. Tissue sections were deparaffinized with EZ Prep (Ventana, Arizona, USA) at 75°C, heat pre-treated in Cell Conditioning 1 (CC1; Ventana, Arizona, USA) using a “standard cell conditioning” protocol for antigen retrieval at 100°C, and then incubated with one drop of inhibitor solution for four minutes at 37°C. Slides were then washed and were incubated for 32 minutes at 37°Cwith anti TLR4 and anti NF-κB (diluted 1:100; Santa Cruz Biotechnology, CA, USA) primary antibody and followed by an incubation with the secondary antibody ultraview universal HRP multimer. The immunolocalized TLR4 proteins were visualized using a copper-enhanced DAB reaction. The slides were counterstained with Hematoxylin II and Bluing Reagent (Ventana, Arizona, USA) for 30 min at 4°C, and then, liquid cover-slip (LCS) was added as a barrier between the aqueous reagents and the air to prevent evaporation, thereby providing sample stability. Samples were dehydrated by sequential washes in graded alcohols: 70% ethanol, 96% ethanol and absolute ethanol and two changes in xylene. Each sample was covered with a small drop of DPX as amounting media. The immuno-stained sections were analyzed by an Olympus BX51 light microscope and DP72 Olympus digital camera (magnification 200X and 400X)(Olympus America Inc, Center Valley, PA, USA).

### Genotyping

TLR4 SNPs (*rs2770150*, *rs10759931*, *rs10759932 and rs4986790*), were genotyped using a TaqMan allelic discrimination assay, as previously described [70]. SNP positional information is described in S2 Table. DNA (10 to 20 ng) from each sample was used in reactions with 5.6 μl of 2X Universal Master Mix and 200 nM primers. All genotypes were determined by endpoint reading using an ABI 7500 real-time PCR machine. The primer and probe mixtures were purchased through the on-demand service of Applied Biosystems. Five percent of the samples were randomly selected and subjected to repeat analysis as a quality control measure for verifying the genotyping procedures.

### Statistical analysis

Genotypic and allelic frequencies were computed and checked for deviation from Hardy–Weinberg equilibrium (http://ihg2.helmholtz-muenchen.de/cgi-bin/hw/hwa1.pl). Case-control and other genetic comparisons were performed using the chi-square test with Yates corrections, and allelic odds ratios (ORs) with 95% confidence intervals (CIs) were calculated with Fisher’s exact test (two-tailed). The statistical analysis was performed using SPSS (Statistical Package for the Social Sciences) 16.0 software for Windows. We considered p values <0.05 as significant. Additionally, Bonferroni’s correction was applied for multiple comparisons.

## Supporting Information

S1 Table Description of primer pairs used in real time PCR reactions. (DOCX)

S2 Table Characteristics of selected polymorphisms involved in the Toll-like receptors 4. (DOCX)

## Abbreviations

TLR4: Toll-like Receptor 4
CRC: colorectal cancer
SNPs: single-nucleotide polymorphisms
LRRs: leucine-rich repeats
DAMP: Pathogen-associated molecular pattern
DAMPs: Damage-associated molecular pattern molecules
TNF: Tumor necrosis factors
TIR: Toll-interleukin1 receptor
NF-κB: Nuclear factor kappa-light-chain-enhancer of activated B cells
MAP: Mycobacterium avium subsp. Para tuberculosis
QPCR: Quantitative PCR
CI’s: Confidence intervals
