# Supplementary material for: Expression and Polymorphism of Toll-Like Receptor 4 and Effect on NF-κB Mediated Inflammation in Colon Cancer Patients
Source: PLoS One. 2016 Jan 15;11(1):e0146333. doi: 10.1371/journal.pone.0146333 (PMC4714746; doi:10.1371/journal.pone.0146333)
Supplement: S1 Table — (DOCX) [file pone.0146333.s001.docx]

**Table 1**: Description of primer sequences used for qRT-PCR reactions

| **Gene** | Primer sequence **(5’ to 3’)** | Amp size  (bp) | Tm  (°C) |
| --- | --- | --- | --- |
| **TLR-4** | Fw: 5'-AATCTAGAGCACTTGGACCTTTCC -3’  Rv 5'-GGGTTCAGGGACAGGTCTAAAGA -3’ | 116 | 60 |
| **IL-1 β** | Fw: 5'-CTGTCCTGCGTGTTGAAAGA-3’  Rv 5'-TTGGGTAATTTTTGGGATCTACA-3’ | 69 | 60 |
| **IL-6** | Fw: 5'-TCTCCACAAGCGCCTTCG-3’  Rv: 5'-CTCAGGGCTGAGATGCCG-3’ | 203 | 60 |
| **IL-8** | Fw: 5'-ATTTCTGCAGCTCTGTGTGAA-3’  Rv: 5'-TGAATTCTCAGCCCTCTTCAA-3’ | 250 | 60 |
| **IL-17** | Fw: 5'-CTCATTGGTGTCACTGCTACTG-3’  Rv: 5' CCTGGATTTCGTGGGATTGTG-3’ | 78 | 60 |
| **GAPDH** | Fw: 5'-GGTATCGTCGAAGGACTCATGAC-3’  Rv: 5'-ATGCCAGTGAGCTTCCCGTTCAGC-3’ | 180 | 58 |
