# Supplementary material for: Expression and Polymorphism of Toll-Like Receptor 4 and Effect on NF-κB Mediated Inflammation in Colon Cancer Patients
Source: PLoS One. 2016 Jan 15;11(1):e0146333. doi: 10.1371/journal.pone.0146333 (PMC4714746; doi:10.1371/journal.pone.0146333)
Supplement: S2 Table — (DOCX) [file pone.0146333.s002.docx]

**S 2 Table**: Characteristics of selected polymorphisms involved in the Toll-like receptors 4

| SNP ID | Chr/ Position | Nucleotide  change | Region | Minor allele frequency (%) | |
| --- | --- | --- | --- | --- | --- |
|  |  |  |  | Cases | Controls |
| **rs2770150** | **Chr 9**/ 117700861 | −3612T>C | 5'Upstream | 0.30 | 0.37 |
| **rs10759931** | **Chr9**/  117701869 | −2604G>A | Promoter | 0.13 | 0.70 |
| **rs10759932** | **Chr 9**/  **120465144** | −1607T>C | Promoter | 0.84 | 0.86 |
| rs**4986790**  Asp299Gly | **Chr 9**/  117713024 | 8552A>G | Exon | 0.04 | 0.05 |
